# Efficacy of an oral and tropically stable lipid-based formulation of Amphotericin B (iCo-010) in an experimental mouse model of systemic candidiasis

**DOI:** 10.1186/1476-511X-12-158

**Published:** 2013-10-29

**Authors:** Fady Ibrahim, Olena Sivak, Ellen K Wasan, Karen Bartlett, Kishor M Wasan

**Affiliations:** 1 Faculty of Pharmaceutical Sciences, The University of British Columbia, 2405 Wesbrook Mall, Vancouver, BC V6T 1Z3, Canada; 2 School of Health Sciences, British Columbia Institute of Technology, 3700 Willingdon Avenue, Burnaby, BC V5G 3H2, Canada; 3 School of Population and Public Health, The University of British Columbia, Vancouver, British Columbia V6T 1Z3, Canada

**Keywords:** Amphotericin B, Lipid-based formulation, Systemic candidiasis, Efficacy, Mice

## Abstract

**Objective:**

An oral lipid based formulation that exhibits tropical stability (iCo-010) was developed to enhance the absorption of orally administered amphotericin B (AmB). iCo-010 has previously shown high efficacy in an acute model of systemic candidiasis in rats, directing the focus of this study to be its efficacy in a chronic model of systemic candidiasis in mice.

**Methods:**

Mice were infected with 0.6 to 1×10^8^ CFUs of *Candida albicans* ATCC 18804 strain by tail vein injection and were left for three days to develop the infection after which time treatment was initiated. The infected animals were assigned to the following treatment groups: no treatment (control) or iCo-010 at 5, 10 and 20 mg/kg administered by oral gavage once daily (QD) for 5 consecutive days. The animals were sacrificed 7 days after the last dose and the concentration of AmB and the fungal burden were assessed within the liver, kidneys, heart, lungs, spleen and brain.

**Results:**

Although the infection was relatively low (~ 60–100 CFUs/ 1 ml tissue homogenate) in the liver, lungs and heart, the infection level was very high (70 000 CFUs / 1 ml tissue homogenate) in the kidney tissues for the control group. The highest concentrations of AmB were recovered in the kidneys and the spleen. The fungal burden in the tissues was lowered by 69-96% in the treatment groups when compared to the control group.

**Conclusion:**

Oral iCo-010 is an effective treatment of systemic candidiasis in the mouse model.

## Introduction

Amphotericin B (AmB) is a polyene macrolide antibiotic used in the treatment of blood-borne fungal infections (systemic candidiasis) and parasitic infections (visceral leishmaniasis). However, its use is hampered by dose-dependent nephrotoxicity and other acute side effects associated with its intravenous administration [1]. In addition, the high cost of the intravenous products and the associated cost of patient hospitalization inhibit the provision of affordable treatment in poor endemic areas located in hot climate zones. Due to these clinical and financial implications the development of an oral AmB formulation is an important endeavor aiming to substitute the present intravenous products [2].

Scientists investigated a wide scope of oral AmB formulations including carbon nanotubes [3,4], cochleate [5-7] and lipid based formulations [8-11]. The carbon nanotubes were tested in a visceral leishmaniasis model in Syrian hamsters and showed high efficacy, however the safety of carbon nanotubes in humans is still questionable [3]. The cochleate formulation was tested in a systemic candidiasis model in mice, but it took 15 days of treatment to lower the infection in the organs [12]. The lipid based formulations of AmB enhanced its solubility and absorption, and reduced its nephrotoxicity [8-11]. However, only one lipid-based formulation (iCo-010) was able to enhance the stability of AmB in tropical conditions [10]. The disposition profile of iCo-010 indicates the accumulation of AmB in the tissues after multiple dosing allowing for therapeutic concentrations to be reached [13,14]. In addition, iCo-010 showed dose dependent efficacy in a rat model of acute systemic candidiasis after only 2 days of treatment [8]. Since rats have no gall bladder and the way they process lipids is different than man [15], studying the efficacy of iCo-010 in mice which have a gall bladder is worth investigation, especially since lipid digestion is a prerequisite for AmB absorption from iCo-010. The objective of this study was to assess the efficacy of iCo-010 formulation in a mouse model of chronic systemic candidiasis infection.

## Materials

Amphotericin B powder, D-alpha-tocopherol polyethylene glycol succinate (TPGS) and 1-amino-4-nitronaphthalene were purchased from Sigma-Aldrich (St Louis, MO, USA). Peceol® (Glycerol monooleates) and Gelucire 44/14® (Lauroyl polyoxylglycerides) were a gift from Gattefossé Canada (Mississauga, Ontario, Canada).

### Experimental methods

#### Preparation of Oral Amphotericin B (iCo-010) formulation

The preparation of iCo-010 was previously described [10,16]. Briefly, AmB, suspended in ethanol, was mixed with Peceol and Gelucire 44/14. VitE-TPGS (5% v/v) was added to obtain a final AmB concentration of 5mg/ml. The mixture was stirred for 1 hour with gentle warming and ethanol was subsequently removed by use of a rotary evaporator.

### Animal study protocol

**Figure 1:**
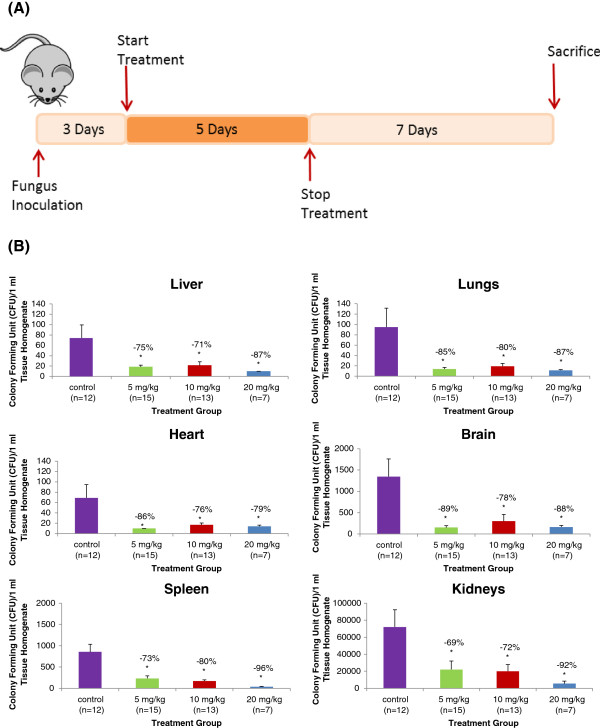
**Inoculation of Candida albicans and treatment protocol of iCo-010 in mice. (A)** Inoculation of *Candida albicans* and treatment protocol of iCo-010 in mice, **(B)** Comparison of the efficacy of oral AmB at 5, 10 and 20 mg/kg in the lipid-based iCo-010 formulation in the liver, lung, heart, brain spleen and kidney tissues of a mouse model of invasive candidiasis. Animals were infected and allowed to establish the infection over two days. The animals were treated for the next five consecutive days once daily and sacrificed 7 days following the completion of the treatment. Organs were harvested and homogenized to count CFU of the fungus/1ml of tissue homogenate (Mean CFU count ±SEM). *p<0.05 significantly different from the untreated control group. The purple column represents the control group, green column represents 5 mg/kg group, red column represent 10 mg/kg group and blue column represents 20 mg/kg group.

All animal study protocols were approved by The University of British Columbia’s Animal Care Committee. Balb C mice (25–30 g body weight) were purchased from Charles River Laboratories (Wilmington, MA) and were kept at controlled temperature and humidity with 12 h light: dark cycles. Each animal was infected with 0.6 to 1×10^8^ CFUs of *Candida albicans* ATCC 18804 strain by tail vein injection. The animals were left for three days to develop the infection after which the treatment was started. The infected animals were assigned to the following groups: no treatment (control) or iCo-010 at 5, 10 and 20 mg/kg administered by oral gavage once daily (QD) for 5 consecutive days with free access to food and water. After the 5 days of treatment, the animals were left for 7 days to recover and then sacrificed (Figure 1A). The right kidney, spleen, liver, heart, brain and lungs were harvested and immediately weighed. The organs were homogenized following the addition of sterile saline solution in a ratio of 1:2 (tissue: saline). An aliquot of the organ homogenate was immediately processed for plating and counting of colony forming units (CFUs) [8].

### High performance liquid chromatography (HPLC) analysis of Amphotericin B

AmB concentrations within tissue samples were determined using a previously developed and published HPLC methods from our laboratory and others [9]. Briefly, 200 μL of tissue homogenate was spiked with 10 μL of internal standard. A double volume of methanol was used to precipitate plasma proteins and to extract AmB into the supernatant after centrifugation at 10000 rpm for 7 min at 15°C. A volume of 90 μL of the supernatant was injected into the HPLC. The analysis was conducted on a BDS Hypersil C18 column (5 μm, 250 × 4.6 mm, Thermo Scientific, Waltham, MA). The lowest limit of quantification was 20 ng/gm.

### Statistical analysis

The difference among the groups was assessed using ANOVA test followed by Tukey’s post-hoc analysis for more than two groups and considering p values of less than 0.05 a significant difference. The percentage of reduction of the fungal burden was calculated using the following formula:

$$\text{The percentage of fungal burden reduction}=\left(\mathrm{CFUs}{\;}_{\mathrm{Cont}}‒\mathrm{CFUs}{\;}_{\mathrm{Treat}}\right)\times 100/\mathrm{CFUs}{\;}_{\mathrm{Cont}}$$

where CFUs is the colony forming units of the fungus per 1 ml of tissue homogenate for the control group (*CFUs*_*Cont*_) and the treated groups (*CFUs*_*Treat*_).

## Results and discussion

While the infection was relatively low (~ 60–100 CFUs/1 ml tissue homogenate) in the liver, lungs and heart, the infection was very high (70 000 CFUs/1 ml tissue homogenate) in the kidney tissues in the control group (Figure 1B). Administration of iCo-010 significantly reduced the CFUs in all the organs as compared to the control. There was no significant difference in the reduction of CFUs in the same organ among the iCo-010 treatment groups. In addition, there was a clear trend of dose response in the kidneys and the spleen. The concentration of AmB in the tissues of the brain and liver were below the limit of quantification of the HPLC assay, in contrast; the highest concentrations of AmB were recovered from the kidneys and the spleen (Table 1).

**Table 1:** **Concentration of AmB (ng/g, Mean +/- SEM) in mouse tissues infected with *Candida albicans *** **7 days after the completion of 5 days oral iCo-010 treatment once daily at 5, 10 and 20 mg/kg doses, (ND; non detectable)**

**Organ**	**AmB concentration (ng/g tissue)**		
	**5 mg/kg (n=15)**	**10 mg/kg (n=13)**	**20 mg/kg (n=5)**
**Kidney**			
	**123.1 ± 16.4**	**323.3 ± 51.5**	494 ± 79.1*
**Liver**			
	**ND**	**ND**	**ND**
**Spleen**			
	**114.5 ± 26.7**	**202.5 ± 15.6**	266.5 ± 29*
**Lung**			
	**ND**	**46.2 ± 1.4**	**78.2 ± 7.5**
**Heart**			
	**ND**	**45 ± 11.6**	**121.4 ± 50.7**
**Brain**			
	**ND**	**ND**	**ND**

*p<0.05 significantly different from the 5 mg/kg treatment group.

The efficacy of iCo-010 in the mouse model of systemic candidiasis is worth investigation since mice possess a gall bladder, which is absent in rats used as the animal model in the previous study [8]. The release of bile secretion from the gall bladder plays an important role in the emulsification and digestion of lipids, the main constituent of iCo-010 [10].

The disease models in the previous and the present study are different. In the present study, the remaining infection was allowed to regrow for 7 days after stopping the treatment allowing for a better estimation of the remaining infection in the tissues. In our preliminary studies, a period of 15 days was chosen as the humane end point as the animals in the control group did not survive or were sacrificed due to severe weight loss after 15 days post inoculation of the fungus. The level of the infection in the organs was significantly reduced in comparison to the control group, which correlates to the administrated doses of iCo-010, most significantly in the spleen and kidney. It is worth noting that the high infection in the kidneys was reduced by 92% even after allowing the infection to regrow for 7 days. This indicates high efficacy of the formulation and high potential for the complete eradication of the infection (Figure 1B).

The remaining concentrations of AmB in the tissues declined throughout the 7 days after the treatment was stopped and were below the quantification limits of the HPLC assay in the liver and the brain. However, the levels in the other tissues were high enough to be quantified (except at the 5 mg/kg dose in the lungs), with the highest recovery of AmB coming from the spleen and the kidneys (Table 1). AmB is known to have high affinity to renal tissues which was previously explained by the high affinity of AmB to plasma LDL, which has high receptor expression in renal tissues [17]. Nonetheless, it is not clear why AmB was highly distributed to the spleen tissues. It is worth noting that the infection in the kidneys and the spleen was the highest among the organs and at the same time both organs had the highest levels of AmB. It is unclear if there is a correlation between the level of infection and the level of AmB in the tissues, where the recruitment of macrophages could play a role in transferring the associated AmB to the infected areas [18,19]. The suppression of infection was significant in all the organs at all dose levels of iCo-010.

## Conclusions

The 5 day treatment of iCo-010 significantly reduced the fungal burden in the mouse organs even at the low dose of 5 mg/kg. The re-grown colonies from 7 days post treatment were 69-96% lower than the control group indicating that the single daily dose of iCo-010 for 5 days is an effective treatment for systemic candidiasis.

## Abbreviations

AmB: Amphotericin B; CFU: Colony forming units; HPLC: High performance liquid chromatography; QD: Once daily.

## Competing interests

The authors declare that they have no competing interests.

## Authors’ contribution

EW prepared the oral AmB formulation, characterized it and was reviewed the manuscript. FI wrote the original draft of the manuscript and completed the AmB tissue distribution analysis. OS completed all the animal studies and reviewed the manuscript. KB prepared the inoculation and reviewed the manuscript. KW wrote and revised the manuscript and was involved in the conceptual design of the studies. All authors read and approved the final manuscript.
